# A Harsh Environment Wireless Pressure Sensing Solution Utilizing High Temperature Electronics

**DOI:** 10.3390/s130302719

**Published:** 2013-02-27

**Authors:** Jie Yang

**Affiliations:** 1 College of Information Science and Engineering, Northeastern University, No. 11, Lane 3, Wenhua Road, Heping District, Shenyang 110819, Liaoning, China; E-Mail: yangjie@ise.neu.edu.cn; Tel.: +86-24-8369-1655; Fax: +86-24-2389-3138; 2 Arkansas Power Electronics International, Inc., 535 W. Research Center Blvd., Fayetteville, AR 72701, USA

**Keywords:** pressure sensor, harsh environment, high temperature, wireless, silicon carbide

## Abstract

Pressure measurement under harsh environments, especially at high temperatures, is of great interest to many industries. The applicability of current pressure sensing technologies in extreme environments is limited by the embedded electronics which cannot survive beyond 300 °C ambient temperature as of today. In this paper, a pressure signal processing and wireless transmission module based on the cutting-edge Silicon Carbide (SiC) devices is designed and developed, for a commercial piezoresistive MEMS pressure sensor from Kulite Semiconductor Products, Inc. Equipped with this advanced high-temperature SiC electronics, not only the sensor head, but the entire pressure sensor suite is capable of operating at 450 °C. The addition of wireless functionality also makes the pressure sensor more flexible in harsh environments by eliminating the costly and fragile cable connections. The proposed approach was verified through prototype fabrication and high temperature bench testing from room temperature up to 450 °C. This novel high-temperature pressure sensing technology can be applied in real-time health monitoring of many systems involving harsh environments, such as military and commercial turbine engines.

## 1. Introduction

Pressure measurement has been one of the primary measurements of interest to engineers and scientists for centuries, since Evangelista Torricelli used a tube of mercury to measure the pressure of air in 1643 [1]. Originally, the pressure was measured by mechanical methods [2–4], which was gradually replaced by electric measurement approaches [5–8]. Due to the continuous progress of micromachining technology in last decades, micro-electromechanical system (MEMS) sensors started playing a major role in pressure measurement [9]. Nowadays there are many types of pressure sensing technologies for different applications, such as capacitive pressure sensors that utilize a diaphragm and a pressure cavity to create a variable capacitance [10–13]; piezoelectric pressure sensors that utilize the piezoelectric effect in some materials to measure the strain caused by pressure [14–18]; surface acoustic wave (SAW) pressure sensors that utilize the phase velocity variation of surface acoustic wave on piezoelectric substrate when pressure is applied [19–23]; optical pressure sensors in which the characteristics of optical signal such as intensity, polarization, phase or spectrum are modulated by the pressure stimulus [24–29]; and the most commonly used piezoresistive pressure sensors, for which the resistance of the piezoresistive material can be altered by the pressure applied on it [30–34].

In industrial applications, many systems that operate at high pressure also suffer from other harsh situations, such as high temperature, high radiation, chemical corrosion, *etc.* Among them, the closest relationship is formed between high temperature and high pressure, as the pressure of gas is directly proportional to the temperature. The requirements of measuring pressure at extreme environments, which are commonly seen in internal combustion engines, gas turbines, oil wells, *etc.*, prompt the development of advanced harsh environment pressure sensing technologies with new materials. Silicon Carbide (SiC) based piezoresistive and capacitive pressure sensors for high-pressure (1,000 psi) measurement at temperatures up to 600 °C have been studied [34–37], while polycrystalline diamond may also be used for high-temperature pressure sensor fabrication [38–39]. However, these types of sensors normally have low signal outputs and require significant embedded signal conditioning electronics. The temperature limitation of silicon electronics is only 150 °C (after special high-temperature treatment), thus the applicability of these sensors in harsh environments is greatly limited. Major pressure sensor manufacturers, such as Honeywell International, Inc., (Morristown, NJ, USA) and Kulite Semiconductor products, Inc. (Leonia, NJ, USA) start implementing and incorporating start-of-art high-temperature silicon-on-insulator (HTSOI) technology into their high-temperature pressure sensors, which elevates the temperature limit of the integrated electronics up to 300 °C [40,41]. In addition, the wired sensors present significant drawbacks in extreme environments, such as the need for electromagnetic shielded wiring harnesses that not only are heavy, bulky and expensive, but also limit the sensor locations. Optical fiber pressure sensing technology is another promising solution for high temperature pressure measurement, because of the small size, light weight, resistance to electromagnetic interference (EMI) and high-temperature capability over 1,000 °C [42–47]. However, similar to the wired sensors, special high temperature housing is required for the optical fibers when it is exposed to high temperatures, with which the flexibility of the sensor is significantly sacrificed. To address the aforementioned problem, passive wireless pressure sensing in harsh environments have also been investigated recently. SAW pressure sensors that are capable of operating in high temperatures up to 800 °C have been reported [48]. Another possible wireless solution is inductive coupling sensors. The inductive coupling sensor is basically a LC resonator including a fixed inductor and a capacitor whose capacitance can be varied according to the pressure. Therefore, the resonating frequency of the LC resonator is pressure-dependent and can be detected remotely by measuring the impedance of an inductively coupled antenna as a function of frequency [49,50]. Passive wireless sensors release the requirements of cable connections and power sources, however, both types of wireless sensors have their drawbacks: SAW sensors are very sensitive to the variation of environmental, geometric and material properties that can usually be seen in harsh environments, while inductive coupling sensors have limited temperature range (below 400 °C), and are inherently prone to EMI.

In this paper a novel harsh environment pressure sensing technology utilizing the high-temperature capability of wide band-gap (band-gap greater than 1.7 eV) electronics is presented. The theoretical temperature limit of wide band-gap materials, such as Silicon Carbide (SiC), Gallium Nitride (GaN) and Aluminum Nitride (AlN) can be as high as 600 °C [51], a significant improvement from silicon and HTSOI electronics. By integrating a SiC high-temperature wireless signal processing and transmission module with the MEMS pressure sensing element, not only the sensor head, but the entire pressure sensor is capable of operating at extreme temperature of 450 °C. The embedded extreme temperature SiC electronics eliminates the need for heavy and costly wire harness and cabling, and yields a highly-integrated and miniaturized sensing system that, along with its wireless functionality, will make it possible to embed such sensing system into complex high-temperature systems (such as turbine engines) without major structural modification.

In Section 2, the design consideration of the proposed SiC signal conditioning and wireless transmission module is introduced. Detailed circuit design of this SiC wireless module is illustrated in Section 3. In Section 4, prototype of the SiC wireless module is presented along with the test results from room temperature to 450 °C, followed by the conclusion and discussion of future works in Section 5.

## 2. Design Consideration of SiC Wireless Module

**Figure 1.**
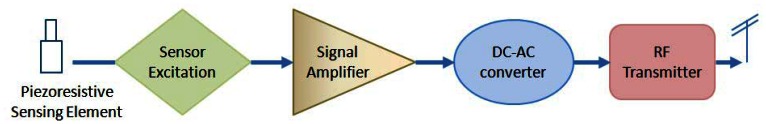
Block diagram of the harsh environment wireless pressure sensing system.

The first step of constructing the harsh environment wireless pressure sensing system is to identify what type of the pressure sensing element should be used. Various high-temperature pressure sensing technologies listed in Section 1 were investigated. Each of these sensing technologies requires different excitation and signal conditioning scheme. Finally, it is determined that the pressure sensing system proposed in this paper is based on the piezoresistive pressure sensing element, mainly for two reasons: (1) the piezoresistive pressure sensing technology is technically more mature compared to other high-temperature sensing technologies, as 500 °C rated pressure sensing elements have been commerically available; and (2) piezoresistive type pressure sensors are easy to be excited and the corresponding signal conditioning circuitry for them is relatively simple. The sceond reason is the determinant, since it is practically very difficult to implement and tune a complex circuit over a broad temperature range. This point will be further discussed in Section 3. The system level design of the proposed SiC wireless sensing module is shown in Figure 1. Besides the sensing element, it contains four major functional circuit blocks: a sensor excitation circuitry, a signal amplifier, a DC to AC converter and a radio frequency (RF) transmitter. The functionalities of these circuit blocks are detailed as follows.

**Figure 2.**
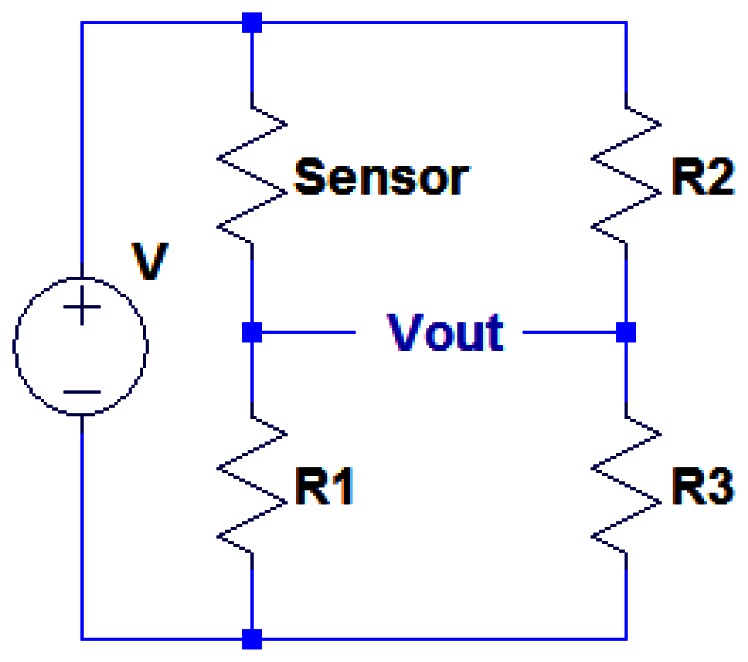
A Wheatstone bridge circuit for excitation of piezoresistive pressure sensing elements.

The first block is a sensor excitation circuitry. For resistive type sensors, it can be as simple as a four-arm Wheatstone bridge as shown in Figure 2. The pressure sensing element is placed in one arm of the bridge and other three arms contain known resistors *R*_1_, *R*_2_ and *R*_3_. The output voltage of this bridge circuit is given by:

(1) $$V_{\text{out}}=\left(\frac{R_{\text{sensor}}}{R_{\text{sensor}}+R_{1}}-\frac{R_{2}}{R_{2}+R_{3}}\right)V$$

The output signal can be set to zero when pressure is not presented, by making *R*_2_ = *R*_sensor_ and *R*_1_ = *R*_3_. The resistance of the sensing element will be changed when a pressure is applied on it, resulting in a voltage output. The signal output is typically low and mixed with surrounding electronic noise. Therefore, a DC-coupled differential amplifier is required to bring the signal strength to a proper level for future processing. Also, for statistic pressure measurement, the output voltage is a slow varying DC-like signal, which cannot be used to directly modulate the RF carrier for wireless transmission. As such, a DC to AC converter has to be utilized to convert the pressure signal into AC format. Finally, a RF transmitter is essential to transmit the pressure signal to the remotely located receiver. Among three commonly used modulation schemes, *i.e.*, amplitude modulation (AM), frequency modulation (FM) and phase modulation (PM), FM is selected for its better noise immunity compared to AM and lower complexity in implementation compared to PM [52,53]. The RF transmitter is consisting of a voltage controlled oscillator (VCO) for generating the RF carrier and performing frequency modulation, and a power amplifier for amplifying the modulated RF signal.

**Figure 3.**
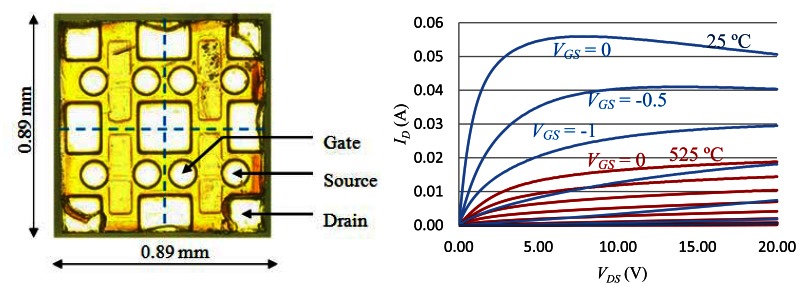
A custom-built Silicon Carbide (SiC) vertical junction field-effect transistor (VJFET) designed for low power radio frequency (RF) applications. Left: The top view of SiC VJEFT bare die. Right: The on-state curves of the SiC vertical JFET.

The key enabling technology of the extreme environment sensing system is the high-temperature wide band-gap electronics, in which the core device is a custom-built SiC n-channel vertical junction field-effect transistor (JFET). This transistor is particularly optimized for high temperature, high frequency and low power consumption operation. It comes in bare die form as traditional packaging approaches would fail at designed operating temperature. This transistor was fully characterized from room temperature to 525 °C using a Signatone RF Probe Station and Sony/Tektronix 372 Curve Tracer. Figure 3(left) shows the top view of the SiC JFET bare die. There are four JFET devices on each die with a common drain terminal. The on-state curves of this transistor at 25 °C and 525 °C are depicted in Figure 3(right) in blue and red, respectively. Furthermore, an empirical full temperature range hyperbolic SPICE model was also built to facilitate the circuit design and simulation [54]. The model can be written as:

(2) $$I_{D}=\beta {\left(V_{GS}-V_{TO}\right)}^{2}(1+\lambda V_{DS})\tanh(\alpha V_{DS})$$

where *I_D_* is drain current; *V_GS_* is gate to source voltage; *V_TO_* is threshold voltage, and *α*, *β* and *λ* are temperature-dependent parameters being optimized from the measured data. The parameters for this particular SiC JFET are:

(3) $$\{\begin{array}{l} \alpha =0.19236\\ \beta =0.01164\\ \lambda =0.0011e^{0.0042268T} \end{array}$$

From Figure 3, it can be clearly seen that some important characteristics of the transistor, such as the transconductance and the threshold voltage, changes drastically with temperature. Besides the SiC JFET, the characteristics of other high temperature active and passive components such as diodes, resistors and capacitors also depend on the ambient temperature. Therefore, special attention has to be paid and temperature compensation techniques are required in high-temperature circuit design.

## 3. Circuit Design of SiC Wireless Module

**Figure 4.**
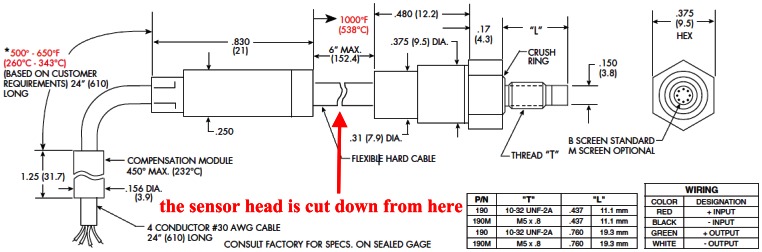
Drawing of the Kulite XTEH-10L-190L pressure transducer from the data sheet of this transducer. The sensor head was cut down from the red mark.

The specific sensing element has to be determined at first before the circuit design of the SiC wireless module is performed, since the signal conditioning block of the SiC wireless module has to be designed based around the output signal of the sensing element. A piezoresistive MEMS pressure sensor from Kulite Semiconductor Products, Inc. (Model No. XTEH-10L-190L) was selected in this project. It is a static pressure transducer with rated temperature of 1,000 °F (538 °C) and rated pressure up to 3,000 psi. The drawing of this transducer is shown in Figure 4 [55]. From Figure 4 it can be realized that the rated temperature is only for the sensor head, while the maximum temperature of the electronics, including the in-line amplifier and the temperature compensation module, is only 450 °F (232 °C). The electronics is saparated from the sensor head by a hard cable. In this work, the pressure transducer was cut down at the hard cable so that only the high-temperature capable sensor head was taken as the signal source.

The biggest challenge of designing this wireless module is that the availability of high-temperature capable active and passive components are very limited, and the characteristics of these circuit components are all temperature-dependent. Since the system should work not only at a particular temperature, but over a very broad temperature range from 25 °C to 450 °C, the design principal is to keep the circuit as simple as possible while achieving all the required functionalities. Simpler design requires fewer components, which will reduce the source of variations and will make circuit tuning much easier over temperatures. It will also help to miniaturize the system and reduce the probability of failure.

**Figure 5.**
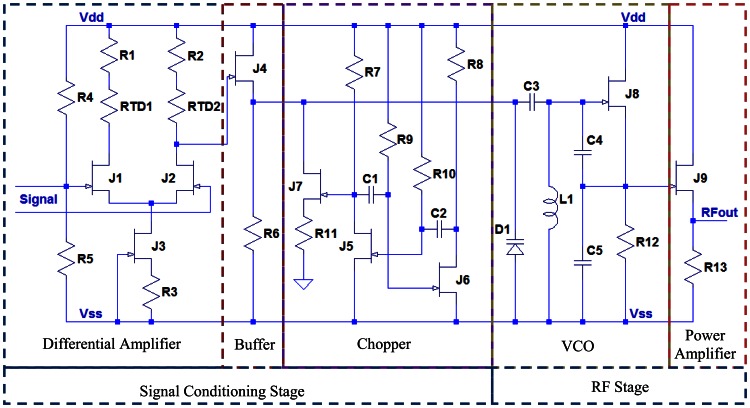
Circuit schematic of SiC wireless sensing module for pressure measurement.

The circuit schematic of the SiC wireless module is depicted in Figure 5. It can be seen that the only transistor being used in the entire system is the n-channel JFET. Since the sensor head has a built-in Wheatstone bridge, sensor excitation circuitry can be neglected here. The schematic of this wireless module contains three major functional blocks. The first block is a differential amplifier with single-ended output. The core of the differential amplifier is a differential pair of two transistors *J*_1_ and *J*_2_, biased by a resistive network including resistors *R*_4_ and *R*_5_, while transistor *J*_3_ and resistor *R*_3_ form a current source structure. Using this current source structure instead of a simple resistor will help to improve the common mode rejection ratio (CMRR), *i.e.*, the ability to attenuate the electrical noise associated with the pressure signal. Since the full-scale output of the sensor head is approximate 100 mV, a differential gain of 10 is desired to amplify the signal strength to 1 V level. The differential gain is proportional to the transconductance of the differential pair and the load resistance. As shown in Figure 3 (right), the transconductance of the SiC JFET drops significantly at 450 °C, resulting in the loss of differential gain at high temperature. The loss can be compensated by attaching resistive temperature detectors (RTD) to the load resistors. The RTD made of platinum is the ideal candidate, due to its higher temperature coefficient of 3,850 ppm (which means better temperature compensation capability) and very linear temperature response from room temperature up to 600 °C.

The next block is a chopper circuit, which is basically a JFET on-off switch driven by a square waveform generated from an astable multivibrator (relaxation oscillator). This astable multivibrator contains two amplifying stage based on transistors *J*_5_ and *J*_6_, which are cross-coupled between drain and gate terminals of each other through capacitive-resistive coupling networks *C*_1_-*R*_9_ and *C*_2_-*R*_10_. When the astable multivibrator is in operation, the states of transistors *J*_5_ and *J*_6_ are alternatively on and off, controlled by the charging and discharging of the capacitors *C*_1_ and *C*_2_. Therefore, two complementary square waveforms are generated at the drain terminals of *J*_5_ and *J*_6_, and either of them can be taken as the output of this astable multivibrator. The oscillating frequency of this astable multivibrator is given by:

(5) $$f=\frac{1}{\ln2\times (R_{9}\times C_{1}+R_{10}\times C_{2})}$$

For the special case of 50% duty cycle, (*i.e.*, transistors *J*_1_ and *J*_2_ have the same on and off time) *R_9_* = *R_10_* = *R* and *C_1_* = *C_2_* = *C*, Equation (5) can be simplified to:

(6) $$f=\frac{1}{2\ln2\times R\times C}$$

When the output of the astable multivibrator is high, the transistor *J*_7_ is on, therefore the output of the amplified pressure signal is bypassed through small resistor *R*_11_. On the contrary, when the output of the astable multivibrator is low, *J*_7_ is off, the pressure signal will be untouched and delivered to the RF stage. Therefore, the output of the chopper will also be a square wave, whose amplitude is the amplitude of the amplified pressure signal, while whose frequency is equal to the frequency of the astable multivibrator. There is also a source follower made of transistor *J*_4_ and resistor *R*_6_, inserted between the differential amplifier and the chopper. The source follower is best known for its high input impedance and low output impedance. Therefore, it can serve as a buffer to interconnect two circuit blocks while preventing signals from unwanted distortion. The differential amplifier, the chopper and the buffer construct the signal conditioning stage of the SiC wireless module.

The last major circuit block is the VCO. It consists of a common-source Colpitts oscillator and a varactor diode *D*_1_. In the Colpitts oscillator, inductor *L_1_* and the series combination of *C*_1_ and *C*_2_ form a parallel resonant tank that determines the frequency of the oscillator. The capacitor *C*_1_ also provides a feedback loop between the gate and source of transistor *J*_8_ to create oscillation. Capacitor *C*_3_ is a coupling capacitor between the low frequency signal and the RF carrier. The RF carrier generated by the Colpitts oscillator is modulated by the square wave pressure signal from signal conditioning stage by means of direct frequency modulation through *D*_1_. This results in the instantaneous frequency deviation about the center carrier frequency being proportional to the amplitude of the modulating signal, and the rate of deviation equal to the frequency of the modulating signal [56]. The instantaneous frequency of the VCO is set by the inductor *L*_1_ and the capacitance combination of capacitors *C_3_*, *C_4_*, *C_5_* and varactor *D_1_* as follows:

(7) $$f=\frac{1}{2\pi }\sqrt{\frac{1}{L_{1}}\left(\frac{C_{3}D_{1}}{C_{3}+D_{1}}+\frac{C_{4}C_{5}}{C_{4}+C_{5}}\right)}$$

As shown in Equation (7), the frequency of the VCO is a function of the capacitance of varactor diode *D*_1_. When an input signal is applied to the varactor, the voltage variation of the pressure signal will change the capacitance of *D*_1_ and will eventually modulate the RF carrier. The modulated signal will be amplified by a power amplifier, which is essentially another source follower made of transistor *J*_9_ and resistor *R*_13_. The power of RF signal is amplified by the source follower via increasing the current of the RF signal. Additionally, this source follower will also serve as a buffer to isolate the VCO from any external loading, as this can shift the frequency of the oscillator in a significant and unpredictable manner [57].

## 4. Prototype and High Temperature Test Results

### 4.1. SiC Wireless Sensing Module Prototype

**Figure 6.**
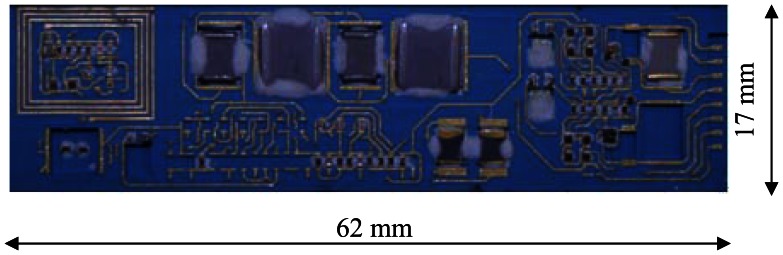
The prototype of SiC wireless module for harsh environment pressure sensing.

The SiC wireless module for pressure sensing system was fabricated using thick-film process on a LTCC substrate. The substrate contains four electric layers, on which the conducting traces were printed using QG150 gold paste from DuPont (Wilmington, DE, USA). A planar inductor for the VCO is also printed on the top layer. The substrate was then populated with SiC JFETs, Platinum RTDs and other high-temperature capable passive components. These components are electrically connected by 0.7-mil gold wires through K&S 4523AD ultrasonic wedge bonder from Kulicke & Soffa Industrial, Inc. (Singapore). Figure 6 shows the fully populated SiC wireless module. There are still some open spaces on the substrate, reserved for tuning resistors and capacitors.

### 4.2. High Temperature Test setup

**Figure 7.**
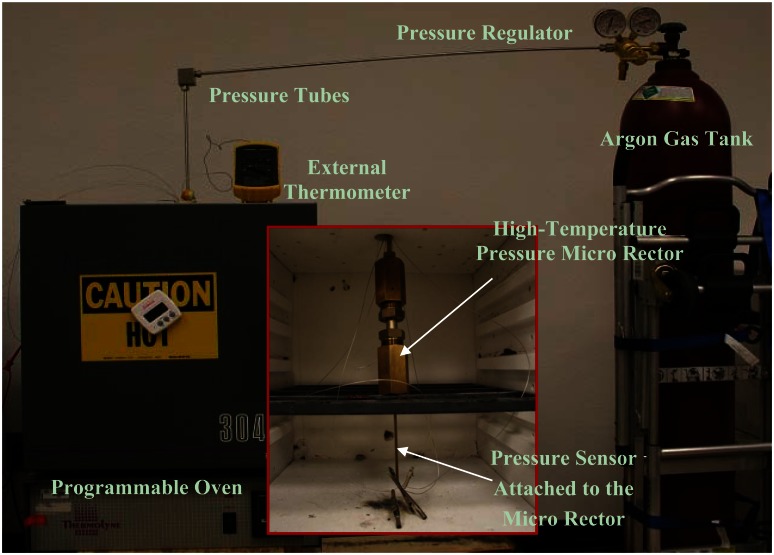
High-temperature test setup for pressure sensor head. Insert: inside view of the oven showing the pressure micro reactor and the sensor head connections.

The high-temperature test setup for wireless pressure measurement is shown in Figure 7. The pressure was provided by an Argon gas tank, with a pressure regulator to control the pressure output. High-temperature pressure tubes were used to connect the pressure regulator to a high-pressure micro reactor. The micro reactor from High Pressure Equipment, Inc. (Erie, PA, USA) is rated up to 60,000 psi and 450 °C. The micro reactor, along with the pressure sensor head attached to it, is placed inside a ThermoLyne 30400 programmable oven. High-temperature anodized aluminum wires were utilized to connect the pressure sensor head with the LTCC prototype board and test instruments located outside the oven. This portion of setup provides the simultaneous high-temperature and high-pressure environments to the pressure sensor head.

**Figure 8.**
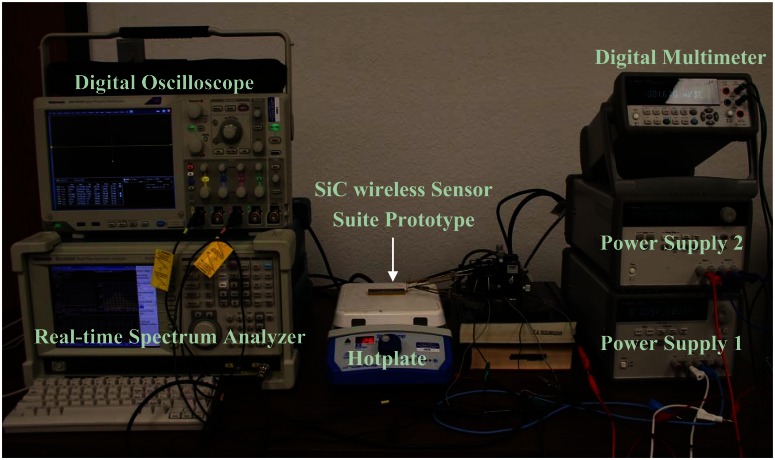
High-temperature test setup for harsh environment SiC pressure sensing wireless module prototype.

The high-temperature test setup for SiC wireless module prototype board is shown in Figure 8. The sensor head was powered by a 10 VDC from a power supply. The output of the sensor head, being monitored by a multimeter, was also fed into the prototype board. The prototype board, powered by the second power supply with ±15 VDC dual voltage outputs, was placed on a hot plate to emulate the high-temperature environment. While the pressure sensor signal and DC power were delivered through connecting probes, the RF signal was picked up from a distance of 10 inches via a whip antenna connected to a Tektronix RSA3303B real-time spectrum analyzer. The received signal was then demodulated directly by the spectrum analyzer to recover the modulating waveform. During the testing, a Tektronix TDS5034B digital phosphor oscilloscope was also utilized to record the output of the signal conditioning stage.

### 4.3. High Temperature Test Results and Analysis

**Figure 9.**
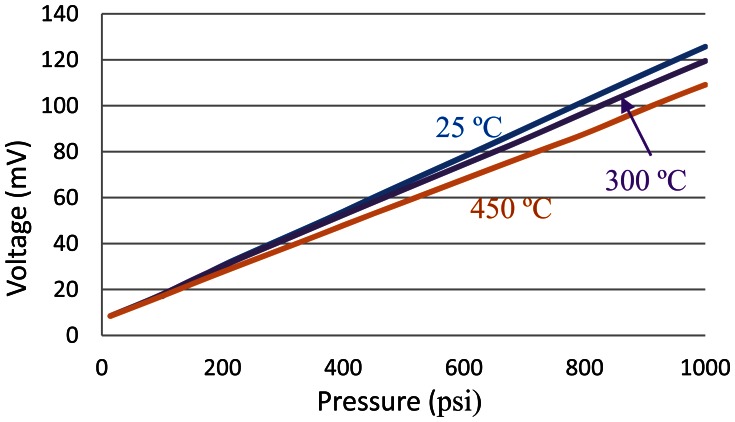
Output of pressure sensor *vs.* applied pressure at various temperatures.

The high-temperature testing of pressure sensor head was performed at first to obtain the response of sensing element at different temperatures. The sensor head was excited using a 10 VDC voltage supply and the output signal was measured using a high-precision digital multilmeter. The output voltage *versus* pressure input at 25 °C, 300 °C and 450 °C are plotted in Figure 9 in blue, purple and orange, respectively. For this XTEH-10L-190L pressure sensor, a very linear relationship between the output voltage and the pressure can be observed at all temperatures. This Kulite pressure sensor also has a very stable temperature performance. The full-scale output of the sensing head reduces from 123 mV at room temperature to 110 mV at 450 °C, a decrease of only approximately 10%. Since the temperature change does not affect the output of the sensor head much, the oven temperature was maintained at 450 °C for the following tests.

**Figure 10.**
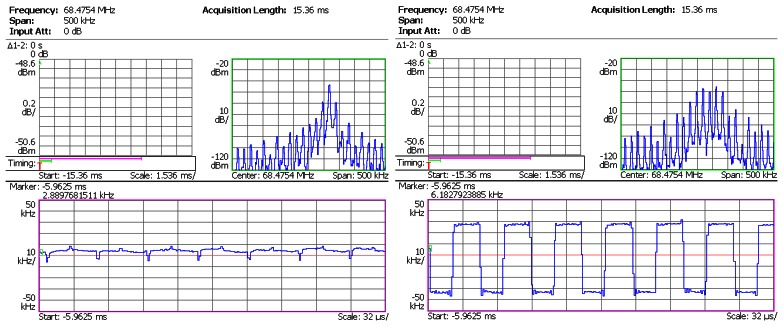
Test result of SiC wireless module prototype at 25 °C. **Left**: at 0 psi; **Right**: at 1,000 psi.

**Figure 11.**
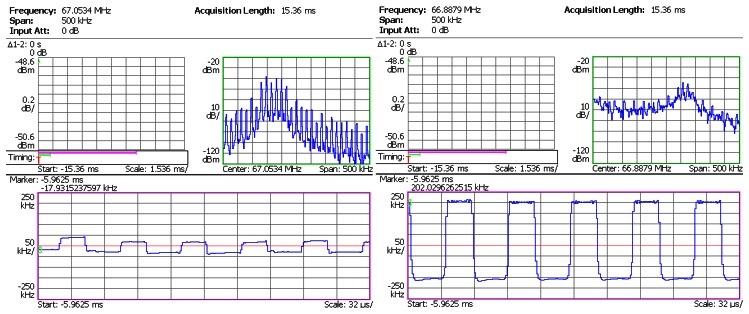
Test result of SiC wireless module prototype at 300 °C. **Left**: at 0 psi; **Right**: at 1,000 psi.

**Figure 12.**
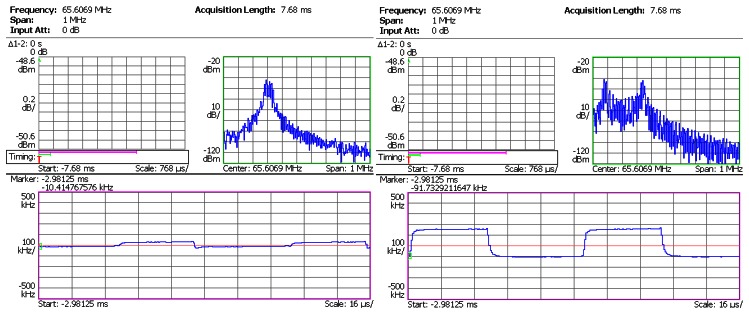
Test result of SiC wireless module prototype at 450 °C. **Left**: at 0 psi; **Right**: at 1,000 psi.

In the next step, the sensor head was connected to the SiC wireless module prototype. The prototype board was heated from room temperature to 450 °C by a hotplate shown in Figure 8. The dwell time at 450 °C was 1 hour. The pressure output from Argon gas tank was regulated in steps up to 1,000 psi. The RF signal from the prototype board was captured by the spectrum analyzer, and was demodulated instantly. The demodulated waveforms from captured RF signal at 25 °C, 300 °C and 450 °C are plotted in Figures 10, 11 and 12, respectively. The upper right subplot in each screen capture from spectrum analyzer (framed in green) shows the close-up view at the peak of RF spectrum. This frequency band is then used by the spectrum analyzer to perform FM demodulation. The bottom subplot (framed in pink) shows the demodulated waveform. A square waveform can be observed throughout the entire temperature range. The shape of this waveform resembles the output of the signal conditioning stage, and the amplitude corresponds to the output of the pressure sensor head. The presence of this waveform indicates that the SiC wireless module prototype successfully acquired the pressure information from the sensor head and wirelessly transmitted it to the remote receiver from room temperature to 450 °C.

**Figure 13.**
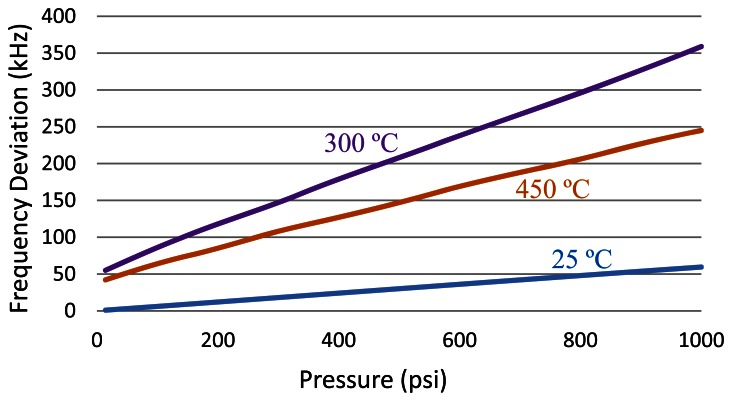
Amplitude of square wave demodulated from RF carrier.

The amplitudes of the demodulated square waveform *versus* supplied pressures at different temperatures are depicted in Figure 13. Since the RF carrier is frequency-modulated, the amplitude of the square waveform is the frequency deviation of the RF carrier measured in Hz. This frequency deviation can be converted into the pressure signal in mV through a system calibration. As shown in Figures 10, 11 and 12, even if the input signal from pressure sensor is not present, a square wave output is still generated by the signal conditioning block. This is due to the SiC device mismatch within the differential amplifier. The SiC device mismatch can be mitigated through circuit tuning, but cannot be completely eliminated across the entire temperature range, unless perfectly matched SiC JFET pairs are available. The presence of a non-zero output for zero input yields an offset that causes an initial frequency deviation, which is temperature dependent. However, it will not affect the pressure measurement as long as the system is fully characterized. From Figure 13, for this particular prototype, the effective full-scale frequency deviation is approximately 60 kHz, 305 kHz and 200 kHz at 25 °C, 300 °C and 450 °C, respectively. The system gain can be defined as:

(5) $$\text{System gain}=\frac{\text{Amplitude of square waveform}\left(\text{kHz}\right)}{\text{Input pressure}(\text{psi})}$$

Higher frequency deviation means higher system gain and better pressure resolution for a given receiver, at the cost of larger bandwidth occupation. The system gain is adjustable based on system requirements. It can be seen that the maximum system gain is obtained in the mid temperature range. Ideally, a constant system gain is preferred. The variation of system gain is mainly due to the inconsistent capacitance change of varactor diode at different temperatures.

## 5. Conclusions and Discussions

In this research work, a high-temperature SiC signal conditioning and wireless transmission module is designed, developed and successful demonstrated of its functionality from 25 °C to 450 °C, in conjunction with a commercial piezoresistive MEMS pressure sensing element. The approach presented in this paper proves the feasibility of embedding the advanced SiC electronics into pressure sensors to expand their operating temperature range. The elevated temperature limit, together with the additional wireless capability, will make it possible to employ this novel sensing technology in real-time health monitoring under extreme environments associated with many mechanical systems, such as aircraft engines and turbine generators.

The immoderate future work in this wireless pressure sensing system is the miniaturization of the wireless module and the integration with sensing elements. The goal is to have both the sensing element and the wireless module co-packaged in a single unit to achieve a highly compact system. Long-term reliability testing of the entire system will also be performed next. Another important aspect of the proposed wireless pressure sensing system is the power source. Current battery technologies could not satisfy the temperature requirement. Depending on the application, there are several possible solutions, including inductive power deliver system, high temperature molten-salt battery and other harsh environment energy scavenging technologies such as vibration based micro-generators or thermal electric generators (TEG). For each of these, a power conditioning circuitry consisting of a voltage regulator and a possible rectifier will be required to provide a stable DC voltage source, which is currently under development.
